# A Grounded Theory Study of Homeopathic Practitioners' Perceptions and Experiences of the Homeopathic Consultation

**DOI:** 10.1155/2011/957506

**Published:** 2010-09-30

**Authors:** Caroline Eyles, Geraldine M. Leydon, George T. Lewith, Sarah Brien

**Affiliations:** Department of Primary Care, University of Southampton, Hampshire SD17 1BJ, UK

## Abstract

Research into the homeopathic consultation has largely focused on patients' experiences, although the practitioner is a crucial component of the therapeutic context and may have an important part in optimizing health outcomes. Therefore the aim of this qualitative research was to gain an in-depth understanding of homeopathic practitioners' perceptions and experiences of the consultation. Medical and non-medical homeopaths were sampled from the registers of the Faculty and Society of Homeopaths. Two phases of data collection were employed. Phase 1 used in depth face-to-face interviews enabling the development of an initial model of the homeopathic consultation. Phase 2 involved observations of homeopathic consultations and practitioner reflective diaries in order to confirm, refute, or enlarge the model. Using the constant comparative method of grounded theory five main categories emerged, *exploring the journey, finding the level, responding therapeutically*, *understanding self*, and *connecting*, forming a model entitled “a theoretical model of a UK classical homeopathic consultation” which describes how homeopaths view and enact the consultation process. This study suggests that the process of identifying and prescribing the remedy is embedded in the consultation, highlighting the interconnectedness of the whole homeopathic consultation and aspects of the consultation that are unique and specific to homeopathy.

## 1. Introduction

 Classical homeopathy [1] is a form of complementary and alternative medicine (CAM) which aims to treat mental, emotional, physical, and spiritual symptoms of the person. During a typically long consultation practitioners ask patients broad questions to elicit subjective symptoms and life experiences [2–4], this enables an understanding of the inner world of the patient and a tool to connect psychological and physiological symptoms [5]. A remedy, based on the patients individual set of symptoms, is then identified and prescribed [6]. Vitalism and holism are two philosophies that are central to the classical homeopathic consultation. Using a vitalistic approach homeopathic practitioners see the purpose of treatment as setting the recovery process in motion by stimulating the patients' self-healing powers or vital force [1, 7]. A holistic approach gives patients an expectation that it will address the cause of their illness [8], enable treatment based on their individual experience [9, 10], and provide a non-reductionist [11, 12] explanatory framework for their illness [13]. Holism also provides the homeopathic practitioner with a means to evaluate the effect of treatment where attention is paid to a wide scope of life experiences and quality of life [14]. 

Homeopathy is a popular CAM [15–18] associated with high patient satisfaction [19–21] and patient perceived positive health outcomes [21, 22]. However, it is a contentious medical approach with debates about the nature of the active ingredient of ultra molecular doses and the mechanism for their action [23, 24], research that suggests that ultra molecular doses exert in vitro effects [25] and the conflicting evidence of efficacy over placebo [26–30]. It has been suggested that the placebo or contextual effects of homeopathic interventions are thought to be as a result of the “therapeutic encounter” that is experienced in the consultation [31–35] and has recently been confirmed in an exploratory clinical trial [36]. If this is the case, identifying the experience and role of both patient and practitioner within the consultation is necessary. 

To date, however, research has largely focused on the patients perspective of the homeopathic consultation, focusing in particular on patient satisfaction [19] and patients views of the consultation [9, 10]. Patients value the quality of the practitioner patient-relationship [37] the holistic approach and being treated as an individual. Patients find these consultations empowering, enabling them to learn more about their own health [19, 38, 39]. They perceive practitioners as being empathic [10] which is perceived by patients as being both therapeutic and supportive [9]. Empathy helps to develop and maintain the therapeutic relationship [10] assists in developing rapport [40] and is related to patient enablement [41] and patient perceived positive change in main complaint and well being [40]. Homeopathic practitioners, compared to general practitioners, demonstrate more empathy by being less neutral with regard to patients opinions, may use disclosure as a means to develop empathy and affiliate themselves with their patients and have a greater ability for showing compassion to their patients [42]. Patients who use CAMs such as homeopathy perceive that through being listened to and heard, a trusting [13], equal and collaborative relationship develops [43] enabling shared decision making [10, 12]. The length of the consultation was also seen as a benefit by patients [9, 19] as they were able to tell their “story” in-depth and have it listened to [10]. 

 Some of the previous literature has described elements of the homeopathic consultation from practitioners' perspectives. Homeopathic practitioners view the consultation as being patient centered [44] and value the long consultations which enable a greater exploration of the patients' symptoms [5]. This exploration is facilitated through the use of narrative competence which can engender hope for the patient [44] and the decision process of identifying a remedy is both cognitive and intuitive [3, 6]. However the consultation is not always perceived by practitioners as harmonious as the therapeutic relationship can be complex and conflicted [45]. 

 Whilst a strong literature has developed on patient views of the homeopathic consultation, homeopathic practitioners' views about their role, experiences and thoughts on the process of how they conduct the consultation are not fully understood. This is important to understand for a number of reasons. Improved understanding of their role may benefit homeopathic practitioners and enhance their clinical outcomes, through improved training and supervision of practitioners and in addition may assist other clinicians through enhanced understanding of components of the consultation that may be generic. 

 The aims of this study were to gain an in-depth understanding of homeopathic practitioners' perceptions and experiences of the classical homeopathic consultation.

## 2. Methods

A qualitative approach was employed in this study using grounded theory. Qualitative methodology is appropriate for in-depth exploration of participants' perceptions and experiences [46]. Grounded theory is suitable for investigating largely unexplored topics, for exploring interactions and for the development of a theoretical model [47–49]. The value of using multiple methodological approaches to explore different perspectives of phenomena has been previously reported [50]. In the field of CAM the validity of qualitative methodology has been identified as fundamental to understanding and describing the philosophical foundations, contextual frameworks and key treatment components of CAM modalities [51, 52]. 

Data collection proceeded through two phases. Phase 1 of the study involved face-to-face interviews with homeopaths from which a tentative theoretical model of the consultation was developed. In phase 2 of the study the model was tested using observations of the consultations and solicited practitioner diaries. The triangulation of different methods to collect data is consistent with theoretical sampling [48] and is a strategy that can increase the robustness of the findings [46]. Approval for Phase 1 of the study was granted by Thames Valley Multi Centre Research Ethics Committee in June 2005 (05/MRE12/42). Approval was also given by 12 Primary Care Trusts. Approval for Phase 2 of the study was granted by Southampton and South West Hampshire Research Ethics Committee (B) in December 2007 (07/H0504/184). All participants provided informed written consent. 

### 2.1. Data Collection and Analysis

**Phase 1**

The aim of phase 1 was to collect data on homeopathic practitioners' experiences and perceptions of the consultation using in-depth face-to-face interviews. 30 participants were identified from the registers of The Society of Homeopaths and The Faculty of Homeopathy and contacted by letter. Initially purposeful sampling enabled a selection of practitioners (see Table 1) who used a range of practice styles. These styles included medical and non-medical homeopaths and homeopaths who worked in private practice, NHS practices and NHS hospitals. The training of these homeopaths also differed between private colleges, university degree courses and faculty training. Practitioners also worked in different locations, including rural and inner city areas. Twenty five of the 30 contacted agreed to be interviewed, reasons for refusal were not provided but the characteristics of non-responders did not appear to be any different from respondents in terms of training, location and clinical experience. In depth interviews were conducted using an open-ended unstructured interview technique, which allowed participants to talk about their experiences of first and subsequent consultations and to illustrate with examples where possible. This allowed participants to express their perspectives on their perceptions, experiences, intentions and roles within the consultation [46, 53]. Analysis was performed concurrently with data collection and as categories emerged from the interviews the questioning became progressively more focused. Theoretical sampling [54] was employed and practitioners were selected because of known aspects of their practice that were likely to contribute to the emerging theoretical model and to negative case analysis. Sampling continued until saturation of the data occurred and no new categories developed [48]. The interviews were recorded for transcription and analyzed by C. Eyles, and the analysis was checked in a sample of interviews by the co-authors, with any disagreements resolved by consensus. Data analysis followed the standard procedure for grounded theory [49]. Initially the data were coded; thereafter concepts and categories were developed from the data and constantly compared and cross-referenced within and between interview transcripts. This process in turn guided data collection and sampling of participants [48, 54]. From the interview data an emerging theoretical model of the homeopathic consultation developed.

**Phase 2**

The aim of phase 2 was to test out aspects of the tentative theoretical model. From this model a checklist of sensitized categories [46, 55] was produced to assist the analysis of data from phase 2. In total 60 homeopaths (members of both the Society of Homeopaths and the Faculty of Homeopathy) were invited to participate in phase 2. Letters were sent to the 25 participants who had been interviewed in phase 1 and 35 new participants were additionally contacted. Participants therefore included both nonmedical and medical homeopaths all of whom were in private practice. NHS homeopathic practitioners were not sampled in phase 2 of the study, due to practical considerations and time constraints. Phase 2 of the data collection process was in two parts, study A and study B.

Study A used non-participant observations of homeopathic consultations [56]; three practitioners took part in study A. Five consultations were observed by C. Eyles and recorded on a camcorder. 2 of these consultations were first consultations and 3 were subsequent consultations. Through observation the contexts within which practitioners operated and interacted were captured. Also any behaviours that may have escaped participants' awareness or that may not have been reported in verbal reports was observed [57]. Observing participants' behaviour in the consultation enabled a clearer understanding of verbal reports and any mismatches between verbal reports of actions and actual actions were identified, and these were then discussed with participants.

**Study B**

Study B involved the completion of solicited practitioner reflective diaries by the participants. Four practitioners took part in study B; and four practitioner diaries were collected in total. The diaries were completed over a two week period using either audio or written format. The practitioners were asked to reflect on their recent consultations, focusing on difficult consultations, using an unstructured narrative format. The diaries allowed exploration into particular aspects of the participants' experience, allowed insight into potentially sensitive areas and into behaviour inaccessible to participant observation and interviews [58–61].

Data from phase 2 of data collection were analyzed using the checklist developed from phase 1. Reflections and observations of actions that confirmed, refuted or provided new data which enriched categories were noted in the checklist and used to inform the final model. The reflections and observations were analyzed by C. Eyles, and S. Brien checked a sample of these.

## 3. Findings

Twenty-five homeopaths were interviewed, 5 consultations were observed and 4 diaries were collected (see Table 1), this was sufficient to achieve saturation. All practitioners practiced classical homeopathy over a period of 3 to 35 years and were based in the south of England. Despite a variation in the sample characteristics there were no overt differences in the process of the consultation between medical and non-medical homeopaths. The main difference between private and NHS homeopaths was found to be the length of the consultation which could vary for a first consultation from 20 minutes for NHS practitioners to 2 hours for private practitioners. A followup consultation could vary from 10 minutes for NHS practitioners to 45 minutes for a private practitioner. 

**Figure 1:**
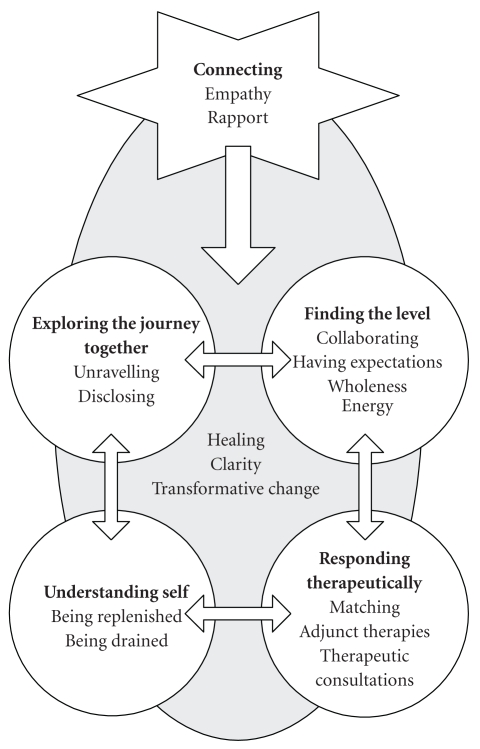
A model of a UK classical homeopathic consultation.

 From the data we present a theoretical model of a UK classical homeopathic consultation from the practitioners' perspectives. Five main categories emerged from the data to form the model; *connecting, exploring the journey, finding the level, responding therapeutically *and* understanding self. Connecting *emerged as the central process and core category in the homeopathic consultation; the other four categories were dependent on and linked to *connecting* (see Figure 1). The intention of the practitioners was to use this process of *connecting *to promote healing for their patients. Each constituent category is described below using illustrative quotations in the indicated tables. Pseudonyms are used to protect the anonymity of participants. 

### 3.1. Core Category 1: Connecting

The practitioners described *connecting* in several ways (see illustrative quotations in Table 2) but it always referred to several factors; the relationship that is formed between the practitioner and the patient, the level of engagement that patients have with homeopathy or holistic consultations, the level of engagement that the practitioner has with practicing homeopathy and the relationship that the practitioner has with themselves. Connecting on these different levels needed to be tailored to the needs of individual patients and tensions could arise between being able to achieve a connection with a patient and over connecting. Practitioners reported, and it was also observed, that they used empathy and rapport building communication strategies to facilitate their connection with patients. In the interviews the meaning of empathy and what was involved in “doing” empathy varied, but all reported that it constituted attentive listening skills with the ability to communicate to the patient that they had understood and heard them. A caring and compassionate demeanour was seen in the observations along with attempts to create rapport; for example, by making the patient welcome and through the skilful mirroring of body language.

### 3.2. Category 2: Exploring the Journey Together

The homeopaths described how the consultation could vary in length, especially the first consultation which could last from 20 minutes to 2 hours. Much of this time was spent listening to the patients story, as was observed in the consultations. Patients would spend the first 20 minutes *disclosing* without interruption and then the homeopath would prompt for further information and *unravel* the patients narrative until an understanding of the patient was reached (see Table 3). Many of the participants referred to homeopathic treatment as being part of a long-term journey of self discovery for both the patient and practitioner as many patients did not know what the underlying reason for their illness was. Therefore, the role of the practitioners at this point of the consultation appeared to be to facilitate the exploration of their symptoms. The process of exploring the patients narrative through their symptoms was described by the practitioners as not only a way for them to connect with their patients, but also as a means of gaining an understanding of the patients beliefs and perspective about their illness. This gathering of symptoms based on the patients subjective experience of their illness was described as purposeful since the intention was to gather enough information and understanding of the patient to prescribe a homeopathic remedy. In classical homeopathy many of the patients' symptoms are relevant to homeopathic prescribing and in particular idiosyncratic symptoms which are integrated and embedded within these stories, making them a source of information for the homeopath. The homeopaths were not only interested in the presenting complaint but also in all idiosyncratic and idiopathic symptoms of the whole person. Such an interest could create a tension for practitioners (and patients) as they attempted to skilfully balance volunteered patient disclosure with purposeful elicitation of information about symptoms. This led many practitioners to either adopt a patient led or more directive style, or even to oscillate back and forth between these formats in order to maintain a balance between the two styles.

### 3.3. Category 3: Finding the Level

Having established a connection and understanding of the patient through exploration of their symptoms homeopaths could then evaluate the patient. This evaluation is sometimes called “case analysis” or “case management” by practitioners [62] and consists of evaluating how to approach treatment for the patient, the patients ability to heal, the extent of their illness, where the focus of their illness lies and how they might respond to treatment (see Table 4). The concepts of *energy*, *wholeness, expectations *and* collaboration *emerged as being important in this evaluative process. The concepts of *energy* (vitalism) and *wholeness* (holism) were often linked by the participants and referred to a process of understanding how individual symptoms could relate to the whole person. These were seen as an approach for connecting different components of the patient, such as the psychological and physical. The homeopaths also used holism and vitalism to evaluate patients' response to treatment; this was described in one of the diary extracts as “Hering's Law of cure” [63]. Hering's Law was described by several practitioners as not focussing on one individual part of the body, but as a reflection of change that flows through the whole person through stimulation of the vitality with homeopathic remedies. The direction of this change will indicate whether the prescription was therapeutic or not and can also be an indicator of the patients' ability to heal. All participants considered that the expectations of both patients and practitioner were important when evaluating the patient. The practitioners' reports revealed that they appeared to engage in a sequential process of assessing, managing, adjusting and matching both patients' and their own expectations. The practitioners construed that these approaches were often new to patients as they may more accustomed to biomedical consultations which may not be as sensitive to patients expectations. It was observed, and the practitioners reported, that they used a collaborative approach which appeared to assist them in utilizing the principles of vitalism and holism and to manage expectations and “socialise patients to holistic consultations”.

### 3.4. Category 4: Responding Therapeutically

Once they have connected only then can the practitioner respond in a therapeutic way to the patient. The responses that the practitioners reported and were observed in the consultation ranged from; the patient can receive benefit from the consultation alone, or, benefit can result from the interaction plus the matching and prescribing of the correctly chosen homeopathic remedy, or change can occur through lifestyle changes (see Table 5). This range of responses is represented by the concepts* therapeutic consultation, matching *and* adjunct therapies. *Several practitioners described occasions where their patients received benefit from a consultation before the administration of a remedy. Some of the practitioners ascribed this benefit to the patient being able to talk and be listened to. Other practitioners enlarged on this by describing how the particular type of exploration of the patients narrative in the homeopathic consultation could lead to the patient making meaningful connections about their illness experiences. However, the majority of practitioners in this study reported that the remedy also had a central role in the homeopathic process. They tended to believe in the power of the homeopathic remedy to heal, either through specific effects and/or through having “*symbolic power”* which may be part of a healing ritual. Finding the right remedy for patients was described by many of the practitioners as a complicated process for which there were several steps. These included a systematic process of deduction, the use of intuition and the use of bodily sensations and awareness to guide remedy choice. Although a homeopathic consultation will typically result in the prescription of a remedy, *adjunct therapies* may also be suggested such as lifestyle changes or referral to another therapy, either in addition to, or, instead of the homeopathic remedy.

### 3.5. Category 5: Understanding Self

Having an understanding of ones self as a professional practitioner was construed to be important by the homeopaths (see Table 6). They reported that it assisted them in the ability to connect and understand their patients and in managing the balance between the challenges and benefits of homeopathic practice. The concepts of *being drained* and *being replenished* respectively represent these challenges and benefits and reveal the tensions and difficulties that the practitioners can encounter in practice. For example, some of the participants felt that their own life experiences contributed to being able to understand others, however there own life experiences can also predispose them to developing preconceptions or assumptions about the patient; and this prior experience was to be used with caution. The practitioners described many experiences that were difficult to manage and contributed to feeling* drained. * Several participants described the feeling of being judged, either by their individual patients or by the scientific community. They revealed that there was a pressure to appear successful in the eyes of the wider world for the sake of the homeopathic profession; some participants framed this within the recent media scrutiny of homeopathy. The sense of being judged could be compounded by the pressure of finding the right remedy for a patient. Meeting the demands of particular patients was also noted as another factor that contributed to difficulties in practice and could also lead the practitioner to become overinvolved emotionally with the patient, resulting in potential health problems for the practitioner. Although all practitioners discussed the difficulties that they experienced many of them described a sense of fulfilment from their practice. This fulfilment derived from occasions when patients would respond positively to a consultation and remedy. Additionally most of the practitioners described various activities that they embarked on in order to balance the demands of practice. Much like any other challenging occupation some of these activities included maintaining hobbies and “outside of work” activities. However, despite these measures two participants, during the course of this study, decided to give up the practice of homeopathy because the apparent challenges outweighed the benefits of continued practice.

## 4. Discussion

This study provides novel qualitative insights into practitioners' experiences and perceptions from which a clear model for the homeopathic consultation has developed. *Connecting* emerged as the core category in the consultation and refers to the relationship that the practitioner forms (or attempts to form) with the patient and themselves as professionals. *Connecting* was crucially linked to and interwoven with other key processes (categories). Through *connecting* a shared journey with the patient was enabled, allowing exploration and evaluation of the patients symptoms which usually involved moving beyond the presenting complaint. Responding to the patient in a therapeutic way could be due to the interaction alone or due to the interaction (including lifestyle changes) within the consultation combined with the homeopathic remedy. Practitioner self awareness was construed as essential for maintaining the balance between the challenges and benefits of practice. 

 The findings of this study build on previous research and broaden our understanding of the homeopathic consultation showing how homeopaths view and enact the process of the whole consultation with their patients. These findings indicate that there are features of the homeopathic consultation that are common to other types of consultation such as counselling and psychotherapy as well as aspects that are unique and specific to homeopathy, this has also been noted elsewhere [64]. Empathy and rapport are common to many therapeutic consultations [65] and previous literature has shown that having a whole person approach [66] being empathic and developing rapport [33, 67] has potential therapeutic value. This study highlights empathy and rapport as skills that were valued and employed by the homeopaths and were viewed as crucial to establishing a relationship with the patient, this is consistent with previous literature [10, 40]. This in turn was seen as assisting patient disclosure and the practitioner in correctly identifying the patients' perspective of their illness and their health needs suggesting that empathy and rapport are important for facilitating all the processes involved in the consultation, although this has been noted in conventional literature [68] this has not been explored in the homeopathic consultation. 

 These findings clearly suggest tensions between the benefits and challenges in the practice of homeopathy not previously known or understood. Although this aspect is discussed and explored by homeopaths in homeopathic journals [69, 70] and books [62, 71] it has not been systematically researched; only one study has looked at the challenges of practice for homeopaths [45]. It is noteworthy that that during the data collection period of this study there was considerable media coverage and scrutiny of homeopathy [72–76]. This may have affected practitioners by highlighting homeopathy's marginalisation from mainstream medicine [77] adding uncertainty and exposing vulnerabilities. This study suggests that homeopaths consider themselves an instrument in the therapeutic process and this has been recognised in conventional medicine as the single most important factor in developing a therapeutic relationship [78–80]. This has not been noted elsewhere in relation to homeopathy. Self awareness and understanding is important as practitioner characteristics can influence the practitioner patient relationship [81, 82]. Some of the participants felt that their own life experiences contributed to being able to understand others. This is consistent with Kleinman's [83] concept of the “wounded healer”. Although this can be a valuable tool for cultivating empathy [65] it can also predispose practitioners to make assumptions about their patients. 

 The collaborative nature of the homeopathic consultation has been noted elsewhere [43] whereby patients are “socialised” to a holistic consultation during which the process of choosing the remedy can be shared with the patient. This sharing of information and choices is similar to shared decision making in the medical consultation [84] which is the ideal model for decision making in the consultation [85]. Our findings add to this as we suggest that the collaborative nature of the homeopathic consultation is also seen in the sequential process of dealing with expectations, which may change in order and according to the patients need. This sequence resembles a process of negotiation that has not been previously noted in the homeopathy literature and is significant given the association between expectations and treatment outcomes observed in medical [86] acupuncture [87] and in homeopathic consultations [88]. 

 The narrative-based approach to the homeopathic consultation which has been previously reported [44, 64] is consistent with other narrative-based therapies [89–92] in that it is concerned with illness experience rather than disease [83]. However there are characteristics of the homeopathic narrative-based approach that are unique and specific to homeopathy [64]. The homeopath probes for specific information that is central to finding the correct homeopathic remedy, such as peculiar and idiosyncratic bodily information, changes in mood, emotional symptoms, sleep and energy symptoms. This indicates that the consultation is significantly different to psychotherapeutic and counselling consultations with which homeopathy is often compared [93]. Moreover the purpose of this narrative exploration is to assist the process of identifying and matching the appropriate homeopathic remedy. This is described in this study as a pattern of decision making which is consistent with the PHIR-M model [6] which includes both cognitive and intuitive processes [3]. The remedy is then prescribed with the intention of treating the whole person including their idiosyncratic and subjective symptoms and thus differs from the medical consultation which fits the drug or intervention to the presenting complaint with the aim of treating the disease. This study adds to this body of knowledge by showing in more detail the process of how the narrative is elicited from the patient and highlights that probing for specific information which may lead to a remedy is central to this process. In this study the practitioners also acknowledged that the benefits of telling a narrative and being listened to and responded to can assist in remedy identification but can also be therapeutic, as “meaning” or “connections” can be constructed through the interaction, this has been previously noted in conventional medical literature [92, 94–96] but not in relation to homeopathy.

 The length of the consultation has been cited as a major reason for homeopathy's popularity [97], despite the variable lengths of the consultation. Depending on the setting; the length of consultations in the NHS can vary from 10 minutes to 1 hour compared to those in private practice which can last from 30 minutes up to 2 hours. Although longer consultations are more likely to result in better health outcomes [98] and contain important elements of care [99] especially in improved recognition and handing of psychosocial problems [100], it is the quality of care that concerns patients [101]. If patients have their emotional needs met, feel listened to and understood regardless of the time spent with the doctor then they are satisfied with the process and the consultation length [102, 103]. Most of the practitioners in this study had consultations that lasted longer than 20 minutes although NHS practitioners in this study and elsewhere [104] have reported that they are able to prescribe homeopathic remedies in a standard 10 minute general practice consultation. Additionally based on the interviews, this study found many similarities between consultations conducted by medical and nonmedical homeopaths and private and NHS practitioners. One of the main differences was that medical homeopaths perceived that they conducted more medically orientated homeopathic consultation which tended to be more concerned initially with the disease process that a patient presented with. Follow-up observational work would confirm this. 

## 5. Implications of This Research

This study has implications for researchers of homeopathy as it demonstrates that the process of finding and prescribing the remedy is embedded in the consultation, highlighting the interconnectedness of the whole homeopathic consultation. The study challenges some assumptions. First, the notion that diagnosis takes place before a research trial; these finding indicate that the homeopath does not make a biomedical diagnosis but understands and evaluates the patients subjective illness according to homeopathic principles. Second, that some non-specific factors such as talking and listening are generic to therapeutic consultations. However, the way in which this is accomplished in the homeopathic consultation is specific to homeopathy. Third, the process of identifying and matching the remedy are specific and integral to the consultation and cannot easily be separated from other non-specific factors such as empathy corroborating a previous theoretical explanation by Weatherley-Jones [105] that the homeopathic remedy is synergistic to the consultation. As such using Whole Systems Research [106] would be a necessary and appropriate research framework to assess this complex intervention.

 This study also has implications for practitioners of homeopathy. The training of homeopathic practitioners has not been systematically researched. The focus of many courses is on the study of homeopathic philosophy, materia medica (remedies) and clinical training, including components that involve interpersonal skills, communication skills, practitioner personal and professional development. For teachers of homeopathy this theoretical model could provide a tool to aid the teaching of interpersonal skills for homeopathic students and for practicing homeopaths a tool to use in supervision.

## 6. Strengths and Limitations

Recruitment for study A (observation of consultations) was challenging as there were many non-responders for this part of the study. When asked homeopaths reported that introducing a third party (camera or researcher) into the consultation would change the dynamics of the interaction, which patients were often paying for. The main limitation of this study was that the observations of the consultations did not include NHS medical homeopaths, this is important as this group of practitioners may not wholly recognise the model as a model of their consultations. However the model was informally shown to several NHS medical homeopaths. These limitations are mitigated by a number of strengths. Triangulation of methods was employed so that the model shows how the practitioners not only viewed but enacted key aspects of the consultation. The model is also likely to be applicable recruiting both medical and nonmedical homeopaths working in variety of locations across different geographical and socioeconomic areas and private and NHS practitioners.

## 7. Conclusion

This study has rigorously explored homeopaths' views on the homeopathic consultation, and in so doing is has highlighted key elements that are unique and specific to it, and the interconnectedness of the processes of identifying and prescribing the remedy. The tendency to label any benefit from homeopathy as placebo effect, nonspecific effects or context effects belies the full range of experiences of the consultation.

##  Funding 

Funding was by National Institute of Health Research (PDA04/CAMs2/02 to C. Eyles).

## Figures and Tables

**Table 1:** Sample characteristics.

	Phase 1: interviews of 30 homeopaths contacted 25 agreed to participate	Phase 2: observations (study A) of 60 homeopaths contacted 3 agreed to participate (5 consultations were observed)	Phase 2: diaries (study B) of 60 homeopaths contacted 4 agreed to participate
Medical homeopaths	12	—	1
Non medical homeopaths	13	3	3
Private practice	15	—	—
NHS practice	3	—	—
NHS & private practice	7	—	—
Female	19	2	4
Male	6	1	—

**Table 2:** Core category 1: connecting.

Subtheme	Examples
Description of connecting	“engaging”, “interface”, “energetic connection”, “relating”, “heart to heart connecting”, “togetherness”, “like a dance”, (Int. Various)
Practitioner connecting to patient	“I donot feel it's been a good consultation unless I've made some sort of connection” (Int 18)
Patient connecting to holistic consultation	“she has been so great to work with because she has really taken on the connection between the mind and the body” (Int 1)
Practitioner connecting to homeopathy	“if I've been in a period where I've been, you know, doing quite a bit of reading and quite a bit of studying of homeopathy, I always feel I enter in with more confidence” (Int 4)
Practitioner connecting to own senses	“If I understand my reactions and if I know that Thuja *(homeopathic remedy)* patients give me a creepy feeling… that might be a very valuable aid to the prescription of Thuja” (Int 20)
Use of empathy	“Listening, showing you care. Understanding what…why… having a relationship with the patient. Understanding why they've come and just being there and sharing that sort of distress in that moment and being open to it I think, it's to do with that really” (Int 11)
Use of rapport	“they've got a nice warm room to sit in something that they are comfortable with so they can find, so its easy to talk to me umm I want to make them feel at ease…” (Int 2)

**Table 3:** Category 2: Exploring the journey together.

Subtheme	Examples
Disclosing	“this is my third consultation with this lady and its only now that she has told me about the death of her daughter” (Diary 1)
Unravelling	“It is delicate at this point I need some answers to some questions and I need to understand, I now need to tread carefully so I donot break her trust or go to fast” (Diary 1)
Joint journey where practitioner facilitates	“They are on a journey and…it's a process of exploration between the practitioner and the patient, sort of discovery…we facilitate and there is joint ownership” (Int 25)
Connecting through exploration	“I looked at her and I just said “how did that make you feel” and she just burst into tears and it all came out…. It was one of those seminal moments in the consultation when suddenly the patients on your side and they've really connected with you” (Int 10)
Patient led exploration	*“*What come out of it is, you know, reveals the patients perception of their illness, not what I'm trying to find out from them, it's them telling me what's the matter with them” (Int 7)
Purposeful exploration	“All we're trying to do is get to know that patient homeopathically to be able to match the picture of the patient problems to a picture of the remedy”. (Int 19)
Directive exploration	“I donot… if this is right…but I'm not interested in wading around the issues. I normally push to go into what's the problem” (Int 14)

**Table 4:** Category 3: finding the level.

Subtheme	Examples
Evaluation of patient	“If we have got on well and the energy is there then I can work out what the patient needs, what we are going to treat, on what level. Am I going to be able to get further than the physical with this patient, can we discover the emotional side…?” (Int 8)
Linking energy and wholeness	“Integrating the whole person through the stimulation of the vital force” “We look at all levels of the person, in an energetic way” (Int 6)
Approach to treatment	“I will need to know how she is emotionally and how she reacts to her environment, memory, her vitality and all that, I need this whole picture to help find a remedy” (Int 1)
Hering's law of cure	“Herings law can give you the confidence that the remedy chosen has acted in a curative manner. So if you see a rash or a discharge the body is pushing symptoms out…” (Diary 2)
Expectations: Assessing	“if you donot know why they have come and they donot know why they have come then how can you know if they are going to get better and what they are expecting from the consultation” (Int 11)
Managing	“I manage their expectations by going through the process and explaining to them what I can or cant do” (Int 12)
Adjusting	“they may think that they want their gout better, and they do…but its all…the gout and the anxiety that matters” (Int 8)
Matching	“we have to agree…its no good me being over confident about what I can do, or them (the patient) wanting too much…” (Int 13)
Collaboration	“but you will have to try not using the antibiotics and see if together we can manage this homeopathically to balance you, what do you think…” (Ob 3)

**Table 5:** Category 4: responding therapeutically.

Subtheme	Examples
Responding through connecting	“I really connect with this patient and more importantly she seems to connect with me. I feel that this is really helpful in finding a good remedy for her, I think this is to do with our mutual understanding” (Diary 1)
Range of responses	“Sometimes it's a remedy… sometimes your response is to watch and wait, sometimes its referral, sometimes its education, sometimes it's you know naturopathic, you know, or nutritional” (Int 25)
Therapeutic consultation	“She always…yes says that she feels better after seeing me and talking. But it's not just that it's, I also think it's that she feels heard really heard” (Int 12)
Benefit through connections	Patient “I noticed that tension makes it worse, its all through my body, its terrible…another connection is that when I eat food I shouldn't eat like wheat…this makes me think about things in my life that I never actually thought before” (Ob 1)
Remedy has specific or symbolic power	“we give *(the remedy)* and say this has power…so its got, as well as any, you know, direct properties that the remedy itself might have on the body, it also has that very symbolic power that it stands for something” (Int 24)
Matching	“finding the right remedy, as we all know, is …is a very complicated task. And I do think that that makes it a sort of exquisite kind of pressure” (Int 24) “The symptoms bring up some possibilities and that will lead me to more questions so there's a successive approximation that happens.” (Int 8) “So sometimes you are drawing on something else arenot you to choose a remedy and it probably is the um intuition in a way” (Int 2) “I am very conscious of just how the patient makes me feel, so you know If I am starting to feel lethargic and increasingly I realise that I am picking this up from the patient and that's another clue to the remedy” (Int 20)
Adjunct therapies	“There's a chiropractor in our clinic, aromatherapist, counselors, physiotherapists…. I've actually got quite a broad range or people to refer on to” (Int 10)

**Table 6:** Category 5: understanding self.

Subtheme	Examples
Understanding self	“how I am in the consultation and what I do is vital, being aware at times of my reactions, as it cannot help but affect how a patient reacts. Surviving practice is about practitioner know thyself and thyself in relation to other people,” (Int 25)
Understanding self helps connecting	“I probably use inner work to make that space feel safe…to develop that energetic connection with the patient” (Int 14)
Prior experience can produce biases	“I am surprised that this lady is not grieving, having lost her mother one year ago…but I realise that I mustn't impose my feelings on this patient” (Diary 1)
Being drained: judged	“You feel that not only are you being judged at a practitioner but to some extent the profession is being judged by our individual successes and failures” (Int 9)
Finding a remedy	“there are more than 4000 remedies now and it can be extremely difficult to find the remedy because obviously if you find the right remedy you get the results” (Int 17)
Challenging patients	“the difficult ones…ummm… needy, aggressive and desperate” (Int 15)
Being too involved	“I can get very drawn into a patients space and you know…I … I think in a lot of occasions this has been quite detrimental to my own health process” (Int 7)
Being replenished: good connections	“Personally I feel like it's a job well done when they've had a good response to a remedy…or made a connection that was valuable for them” (Int 1)
Hobbies	“I do it through music. I'm a jazz singer and I… cos I try and sing every day and I feel better” (Int 6)
supervision	“I see a counsellor, for my own development…I also do back to back mentoring with another homeopath…and I also go to group peer supervision and one to one supervision” (Int 23)
